# Single nucleotide polymorphisms rs701848 and rs2735343 in PTEN increases cancer risks in an Asian population

**DOI:** 10.18632/oncotarget.22019

**Published:** 2017-10-24

**Authors:** Dan-Dan Song, Qian Zhang, Jing-Hua Li, Rui-Min Hao, Ying Ma, Ping-Yu Wang, Shu-Yang Xie

**Affiliations:** ^1^ Key Laboratory of Tumor Molecular Biology in Binzhou Medical University, Department of Biochemistry and Molecular Biology, Binzhou Medical University, Yantai, ShanDong 264003, P.R.China; ^2^ Department of Epidemiology, Binzhou Medical University, Yantai, ShanDong 264003, P.R.China

**Keywords:** PTEN, SNP, cancer susceptibility, meta-analysis

## Abstract

We performed this meta-analysis to analyze the cancer risk to individuals carrying the rs701848 and rs2735343 single nucleotide polymorphisms (SNPs) in the phosphatase and tensin homolog (*PTEN*) gene. We searched the PubMed, EMBASE, Cochrane library and the national knowledge infrastructure of China (CNKI) databases and identified 18 eligible case-control studies with 5458 cases and 6003 controls for rs701848 as well as 5490 cases and 6209 controls for rs2735343. Our analyses demonstrated that cancer risk was associated with rs701848 in the recessive model (CC vs. CT+TT, OR=1.169, 95% CI: 1.061-1.288) and with rs2735343 in the dominant model (GC+CC vs. GG, OR=0.758, 95% CI: 0.590-0.972). Subgroup analysis showed that in Asian subjects, carrying the C allele of rs701848 or GG genotype of rs2735343 was associated with increased cancer risk. Moreover, Asian subjects carrying the TC/CC genotype or C allele of rs701848 were associated with increased risk of esophageal squamous cell cancer. This meta-analysis indicates that the *PTEN* rs701848 (CC) and rs2735343 (GG) polymorphisms are associated with increased cancer risk in Asian subjects.

## INTRODUCTION

Phosphatase and tensin homolog (*PTEN*) is also known as mutated in multiple advanced cancers 1 (*MMAC1*) or TGF-β regulated and epithelial cell-enriched phosphatase 1 (*TEP1*) and is a tumor suppressor gene [1–3]. It is located on human chromosome 10q23 and encodes a 403 amino acid protein that is associated with lipid and protein associated phosphoinositide 3-phosphatase activity. PTEN is generally cytosolic and regulates phosphatidylinositol 3,4,5-trisphosphate (PIP3) levels; small fraction of PTEN is recruited to the plasma membrane [4]. PTEN reduces PIP3 levels [5], which decreases mTOR/AKT signaling pathway that is critical for cancer cell growth, survival and progression [6, 7].

Single-nucleotide polymorphisms (SNPs) are the most common type of genetic variations that involve change in a single nucleotide in a gene or associated genetic elements, which affect gene expression [8]. A number of SNPs have been implicated in various human diseases [9–13] and are clinically relevant as factors that determine cancer susceptibility, prognosis of survival, and treatment response [8]. A number of SNPs, mutations and deletions in *PTEN* have been reported in many human cancers including glioblastoma [14–19].

The relationship between cancer risk and two *PTEN* SNPs, rs701848 and rs2735343 is controversial. The rs701848 SNP is associated with increased risk of breast cancer (BC) [20], renal cell cancer (RCC) [21], colorectal cancer (CRC) [22], and esophageal squamous cell cancer (ESCC) [23]. However, there are contradictory reports that show no correlation between rs701848 and the risk of ESCC [24] and hepatocellular carcinoma (HCC) [25]. Moreover, rs2735343 is associated with increased breast cancer risk in early onset and familial cases [26]. Subjects with rs2735343 (GG) are associated with elevated risk of ESCC [23]. However, there is no association between rs2735343 (G/C) and the risk of endometrial cancer [27]. In this meta-analysis, we estimated the association between cancer susceptibility and the *PTEN* SNPs, rs701848 and rs2735343.

## RESULTS

### Literature search and eligibility criteria

**Figure 1:**
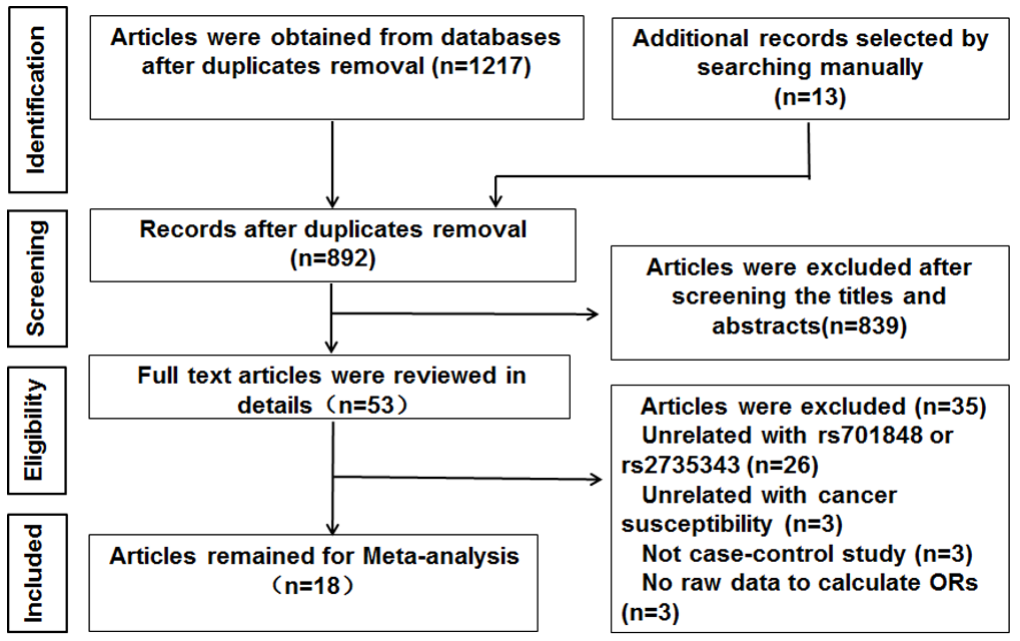
Flow diagram of study selection process

We searched the PubMed, EMBASE, Cochrane library and the national knowledge infrastructure of China (CNKI) databases and identified 1230 articles. After removing the duplicate articles, 892 articles still remained for further evaluation. Then, we reviewed article titles and abstracts and excluded 839 reports that were not related to cancer risk and *PTEN* SNPs. We then assessed the remaining 53 reports in greater detail and excluded 35 articles that did not satisfy the eligibility criteria. Finally, 18 eligible case-control studies were included in our meta-analysis [20–37] (Figure 1, Table 1). Moreover, we analyzed the data of each SNP independently in studies that investigated both rs701848 and rs2735343 SNPs [23, 24, 32, 33]. Overall, we analyzed 5458 cases and 6003 controls for rs701848 in 14 studies as well as 5490 cases and 6209 controls for rs2735343 in 8 studies.

**Table 1:** Characteristics of studies on the associations between rs701848(C/T) and rs2735343(C/G) polymorphisms in PTEN and cancer

Author	Year	Country	Ethnicity	Cancer type	Genotyping method	Source of controls	P value for HWE ^q^	Case/control	Frequency distributions of the genotypes					
									Case (n)			Control (n)		
rs701848									TT	TC	CC	TT	TC	CC
Li	2017	China	Asian	BC **^c^**	TaqMan **^l^**	HB **°**	0.22559	880/910	215	468	197	273	474	163
Lin	2015	China	Asian	CRC **^d^**	TaqMan	HB	0.32525	780/764	186	421	173	229	397	138
Xu	2015	China	Asian	ESCC **^e^**	TaqMan	PB **^p^**	0.19999	425/446	205	182	38	243	182	21
Jing	2014	China	Asian	CRC	SNPscan **^m^**	PB	0.26281	519/537	190	253	94	162	272	85
Jang	2013	China	Asian	ESCC	PCR-RFLP **^n^**	PB	0.0306	304/413	91	155	58	183	165	65
Ma	2012	China	Asian	ESCC	PCR-RFLP	PB	0.20173	226/226	70	121	35	103	90	33
Cao	2012	China	Asian	RCC **^f^**	TaqMan	HB	0.52099	710/760	222	338	150	277	351	132
Chen	2012	China	Asian	PC **^g^**	TaqMan	HB	0.81281	666/708	212	329	125	235	353	120
Ding	2011	China	Asian	HCC **^h^**	PCR-RFLP	PB	0.32694	131/215	43	67	21	65	116	34
Hiroshi	2009	Japan	Asian	PC	PCR-RFLP	HB	0.51513	140/167	51	58	31	47	90	30
Song	2009	China	Asian	LC **^i^**	PCR-RFLP	HB	0.92453	149/104	46	74	29	26	54	24
Liu	2009	China	Asian	GC **^j^**	PCR-RFLP	HB	0.92453	58/104	17	35	6	24	54	26
Liu	2008	China	Asian	LC	PCR-RFLP	HB	0.92453	91/104	29	45	17	26	54	24
Rajaraman	2007	American	Mixed-race **^a^**	Glioma	TaqMan	HB	0.98643	379/545	138	184	57	190	262	93
rs2735343									GG	GC	CC	GG	GC	CC
Chen	2016	China	Asian	BC	SNPscan	HB	0.53023	728/669	190	360	178	142	348	179
Jang	2013	China	Asian	ESCC	PCR-RFLP	PB	0.07336	304/413	108	151	45	93	181	139
Ma	2012	China	Asian	ESCC	PCR-RFLP	PB	0.38422	226/226	71	117	38	45	100	81
Slattery	2012	Mexico American	Mixed race **^b^**	BC	multiplexed bead array assay	PB	-	3590/4183	1398	2192*	-	1491	2692*	-
Lacey	2011	Poland	Caucasian	EC **^k^**	Infinium assay	PB	0.47144	416/406	211	163	42	215	154	37
Song	2009	China	Asian	GC	PCR-RFLP	HB	0.51145	58/104	4	33	21	30	57	17
Shi	2009	China	Asian	Lung cancer	PCR-RFLP	HB	0.54184	77/104	32	37	8	29	57	18
Liu	2008	China	Asian	LC	PCR-RFLP	HB	0.54184	91/104	29	46	16	18	57	29

^a^ Mixed-race consists of White, non-Hispanic, Hispanic, and Black; ^b^ Mixed-race consists of Hispanic, native American, and NHW (non-Hispanic white) women; ^c^ BC, breast cancer; ^d^ CRC, colorectal cancer; ^e^ ESCC, esophageal squamous cell carcinoma; ^f^ RCC, renal cell carcinoma; ^g^ PC, prostate carcinoma; ^h^ HCC, hepatocellular carcinoma; ^i^ LC, laryngocarcinoma; ^j^ GC, gastric cancer; ^k^ EC, endometrial cancer ; ^l^ TaqMan, TaqMan SNP Genotyping Assays; ^m^ SNPscan, SNPscanGenotyping system; ^n^ PCR-RFLP, Polymerase chain reaction (PCR)–restriction fragment length polymorphism assays; ° HB, hospital-based; ^p^ PB, population-based; ^q^ HWE, Hardy-Weinberg equilibrium; * The number of GC/CC is 2192 in cases, 2692 in controls.

### Study characteristics

**Figure 2:**
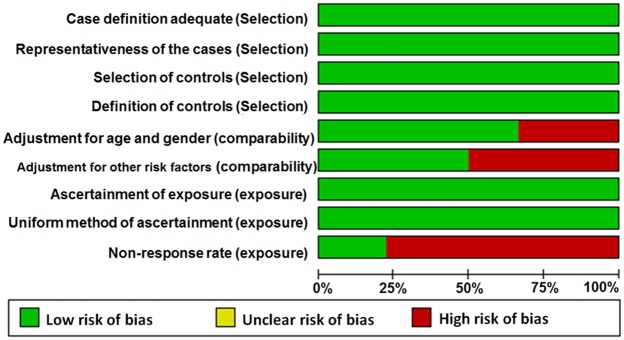
Quality assessment scale of eligible studies

Table 1 summarizes the main characteristics of the included studies such as first author, published year, country of origin where the study was conducted, ethnicity, cancer type, genotyping method, source of controls, and frequency distributions of the genotypes for cases and controls (Table 1). Among the 18 studies, 14 were conducted in China and 1 each in Japan, USA, Poland, and Mexico/USA. Overall, 15 out of 18 studies enrolled Asian subjects, 1 study enrolled Caucasian individuals, and 2 studies enrolled subjects from mixed races. The cancer types that were analyzed in these studies included colorectal cancer (CRC), esophageal squamous cell carcinoma (ESCC), hepatocellular carcinoma (HCC), renal cell carcinoma (RCC), prostate carcinoma (PC), laryngocarcinoma (LC), gastric cancer (GC), breast cancer (BC), glioma, and endometrial cancer (EC). PTEN genotyping was performed by Taqman (6 studies), Polymerase chain reaction–restriction fragment length polymorphism (PCR-RFLP; 8 studies), SNPscan (2 studies), Infinium assay (1 study) and multiplexed bead arrays (1study). Among the 18 studies, 10 were hospital-based (HB) and 8 were public-based (PB). In 16 out of 18 eligible studies, genotype distributions of rs701848 and rs2735343 in the controls were in agreement with Hardy-Weinberg equilibrium (HWE). The P value for Jang's study [23] was less than 0.05, whereas there was no available data to calculate P value for HWE in Slattery's study [28]. The genomic DNA was isolated from blood samples in 17 out of 18 included studies, whereas in Slattery's study [28] whole blood or mouthwash samples were used for isolating genomic DNA. The quality scores according to Newcastle-Ottawa quality assessment scale varied 6 to 9 in the 18 studies (Figure 2, Supplementary Table 1).

### Association between *PTEN* SNPs and cancer risk

**Figure 3:**
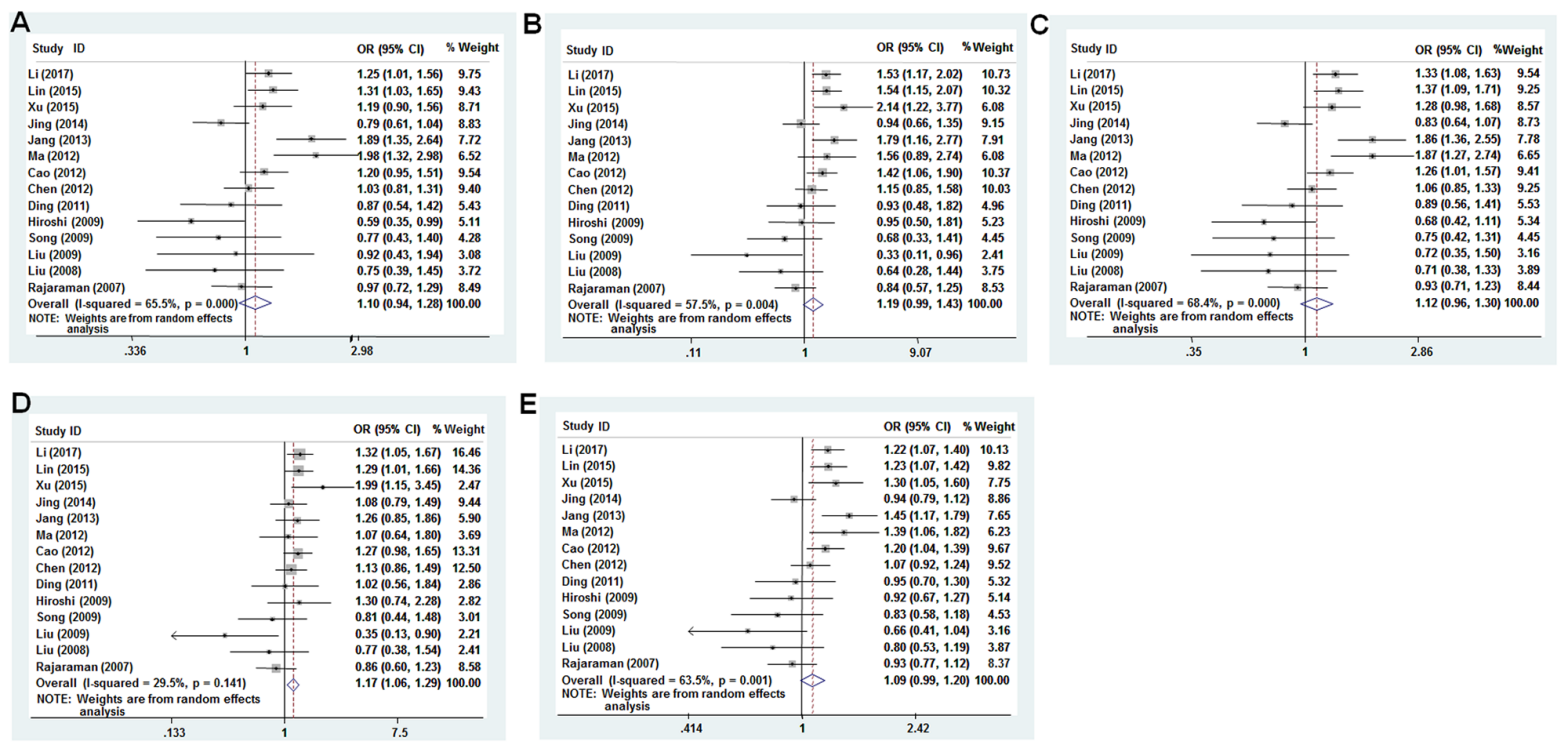
Forest plot of cancer risk associated with rs701848 (T>C) models **(A)** heterozygous model; **(B)** homozygous model; **(C)** dominant model; **(D)** recessive model; **(E)** additive model.

**Figure 4:**
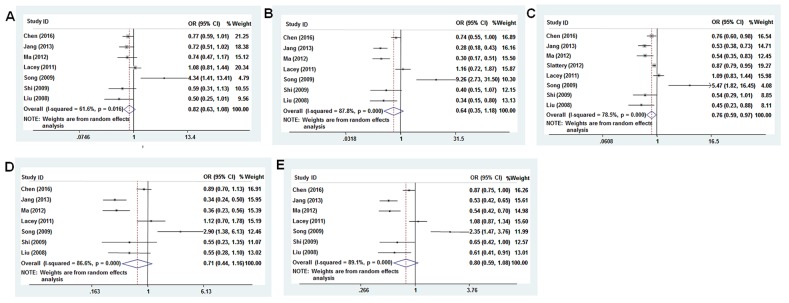
Forest plot of cancer risk associated with rs2735343 (G>C) models **(A)** heterozygous model; **(B)** homozygous model; **(C)** dominant model; **(D)** recessive model; **(E)** additive model.

We analyzed the association between the *PTEN* SNPs and cancer risk using dominant, recessive, heterozygous, homozygous, and additive models. The rs701848 CC genotype was associated with 1.169-fold increased cancer risk in recessive model (OR = 1.169, 95% CI: 1.061-1.288, Table 2, Figure 3D). However, it was not associated with cancer risk in heterozygous (OR = 1.099, 95% CI: 0.943 - 1.280), homozygous (OR = 1.190, 95% CI: 0.990 - 1.432), dominant (OR = 1.115, 95% CI: 0.959 - 1.297) and additive (OR = 1.088, 95% CI: 0.990 - 1.196) models (Table 2, Figure 3A-3C, 3E). The rs2735343 GG genotype showed increased cancer risk in the dominant model (OR = 0.758, 95% CI: 0.590 - 0.972, Table 2, Figure 4C). The rs2735343 GG polymorphism was not associated with cancer risk in heterozygous (OR = 0.821, 95% CI: 0.625 - 1.079), homozygous (OR = 0.642, 95% CI: 0.349 - 1.180), recessive (OR = 0.711, 95% CI: 0.437 - 1.156), and additive (OR = 0.802, 95% CI: 0.594 - 1.083) models (Table 2, Figure 3A, 3B, 3D, 3E).

**Table 2:** ORs and 95% CI for cancers and rs701848 or rs2735343 polymorphism in PTEN under different genetic models

Genetic models	n	OR (95% CI)	*P* (OR)	Model (method)	I-square (%)	*P* (H)	*P* (Begg)	*P* (Egger)
***rs701848***								
Heterozygous model (TC *vs* TT)	14	1.099(0.943,1.280)	0.226	R	65.5	0.000	0.228	0.305
Homozygous model (CC *vs* TT)	14	1.190(0.990,1.432)	0.064	R	57.5	0.004	0.037	0.054
Dominant model (TC+CC *vs* TT)	14	1.115(0.959,1.297)	0.157	R	68.4	0.000	0.274	0.154
Recessive model (CC *vs* CT+TT)	14	1.169(1.061,1.288)	0.002	F	29.5	0.141	0.012	0.060
Additive (C *vs* T)	14	1.088(0.990,1.196)	0.080	R	63.5	0.001	0.101	0.066
***rs2735343***								
Heterozygous model (GC *vs* GG)	7	0.821(0.625,1.079)	0.157	R	61.6	0.016	0.764	0.800
Homozygous model (CC *vs* GG)	7	0.642(0.349,1.180)	0.154	R	87.8	0.000	0.368	0.796
Dominant model (GC+CC *vs* GG)	8	0.758(0.590, 0.972)	0.029	R	78.5	0.000	1.000	0.614
Recessive model (CC *vs* GC+GG)	7	0.711(0.437,1.156)	0.169	R	86.6	0.000	0.548	0.974
Additive (C *vs* G)	7	0.802(0.594,1.083)	0.150	R	89.1	0.000	0.368	0.909

OR, odds ratio; CI, confidence intervals; *P* (OR), *P* for heterogeneity; *P* (H), *P* for heterogeneity; n, number of included studies; R, random-effect model; F, fixed-effect method.

The pooled odds ratio (OR) for rs701848 polymorphism in recessive model was analyzed by fixed-effects model. Since data was heterogeneous, random effects model was used to analyze the significance of pooled OR for rs701848 in homozygous, heterozygous, dominant, and recessive models and in all models for rs2735343 (Table 2).

### Subgroup analysis

We performed subgroup analysis based on ethnicity, cancer type, source of controls, genotyping methods, quality score (at the median cut-off point of 8), and sample size, respectively. We observed that Asian individuals with rs701848 C allele were associated with 1.105-fold increased cancer risk than the non-Asian population (C vs T, OR = 1.105, 95% CI: 1.003 - 1.217, Table 3). The CC genotype showed 1.234-fold and 1.185-fold higher cancer risk respectively in homozygous (CC vs TT, OR = 1.234, 95% CI: 1.024 - 1.488) and recessive (CC vs CT+TT, OR = 1.185, 95% CI: 1.049 - 1.338, Table 3) models in the Asian population. Asian individuals with rs2735343 GG genotype were also associated with increased risk of cancer (Table 4). Regarding cancer types, the rs701848 CC genotype showed 1.813-fold increased ESCC risk than the rs701848 TT genotype (CC vs. TT, OR = 1.813, 95% CI: 1.352 - 2.433, Table 3). There was no association between *PTEN* SNPs and cancer risk in all models of rs701848 in regard to hospital or public based studies (Table 3).

**Table 3:** Subgroup analyses of rs701848 polymorphism in PTEN with cancer risk

Subgroups		TC *vs* TT			CC vs TT			TC+CC *vs* TT			CC *vs* CT+TT			C *vs* T		
		N	OR (95% CI)	*P* (OR)	N	OR (95% CI)	*P* (OR)	N	OR (95% CI)	*P* (OR)	N	OR (95% CI)	*P* (OR)	N	OR (95% CI)	*P* (OR)
Ethnicity	Asian	13	1.110 (0.942, 1.308)	0.214	13	1.234 (1.024, 1.488)	0.027	13	1.132 (0.965, 1.329)	0.128	13	1.185 (1.049, 1.338)	0.006	13	1.105 (1.003, 1.217)	0.043
	Non-Asian	1	0.967 (0.724, 1.291)	0.820	1	0.844 (0.568, 1.254)	0.401	1	0.935 (0.711, 1.229)	0.628	1	0.860 (0.601, 1.232)	0.412	1	0.928 (0.768, 1.122)	0.441
Cancer type	ESCC	3	1.609 (1.140, 2.272)	0.007	3	1.813 (1.352, 2.433)	0.000	3	1.612 (1.240, 2.096)	0.000	3	1.358 (0.982, 1.880)	0.065	3	1.375 (1.206, 1.567)	0.000
	Other	11	1.004 (0.874, 1.154)	0.952	11	1.077 (0.881, 1.315)	0.471	11	1.014 (0.874, 1.176)	0.855	11	1.113 (0.972, 1.274)	0.120	11	1.026 (0.930, 1.133)	0.606
Source of control	PB	5	1.249 (0.863, 1.808)	0.238	5	1.387 (0.983, 1.959)	0.063	5	1.270 (0.902, 1.788)	0.171	5	1.207 (0.988, 1.476)	0.066	5	1.187 (0.979, 1.438)	0.081
	HB	9	1.060 (0.919, 1.222)	0.425	9	1.097 (0.870, 1.383)	0.433	9	1.060 (0.905, 1.242)	0.470	9	1.106 (0.938, 1.304)	0.229	9	1.043 (0.933, 1.166)	0.461
Genotyping	mic-Array	7	1.103 (0.972, 1.252)	0.129	7	1.293 (1.061, 1.576)	0.011	7	1.144 (0.997, 1.314)	0.056	7	1.214 (1.065, 1.384)	0.004	7	1.123 (1.023, 1.232)	0.015
	PCR-RFLP	7	1.040 (0.698, 1.552)	0.846	7	0.964 (0.648, 1.435)	0.857	7	1.013 (0.691, 1.484)	0.948	7	0.986 (0.766, 1.270)	0.913	7	0.996 (0.795, 1.248)	0.973
Sample size	<500	6	0.924 (0.614, 1.391)	0.706	6	0.852 (0.583, 1.246)	0.409	6	0.900 (0.617, 1.314)	0.586	6	0.912 (0.683, 1.218)	0.532	6	0.928 (0.751, 1.148)	0.493
	≥500	8	1.161 (0.996, 1.354)	0.057	8	1.337 (1.109, 1.613)	0.002	8	1.202 (1.029, 1.403)	0.020	8	1.219 (1.085, 1.369)	0.001	8	1.151 (1.046, 1.267)	0.004
Quality score	<8	6	0.920 (0.720, 1.176)	0.506	6	0.851 (0.565, 1.281)	0.439	6	0.897 (0.687, 1.171)	0.425	6	0.936 (0.684, 1.280)	0.678	6	0.923 (0.762, 1.117)	0.410
	≥8	8	1.213 (0.996, 1.478)	0.054	8	1.367 (1.142, 1.636)	0.001	8	1.248 (1.038, 1.501)	0.018	8	1.225 (1.087, 1.380)	0.001	8	1.175 (1.061, 1.300)	0.002

**Table 4:** Subgroup analyses of rs2735343 polymorphism in PTEN with cancer risk

Subgroups		GC *vs* GG			CC *vs* GG			GC+CC *vs* GG			CC *vs* GC+GG			C *vs* G		
		N	OR (95% CI)	*P* (OR)	N	OR (95% CI)	*P* (OR)	N	OR (95% CI)	*P* (OR)	N	OR (95% CI)	*P* (OR)	N	OR (95% CI)	*P* (OR)
Ethnicity	Asian	6	0.765 (0.565, 1.036)	0.083	6	0.577 (0.292, 1.139)	0.113	6	0.684 (0.468, 1.001)	0.050	6	0.658 (0.379, 1.141)	0.136	6	0.760 (0.543, 1.064)	0.110
	Non-Asian	1	1.079 (0.806, 1.443)	0.611	1	1.157 (0.715, 1.871)	0.553	2	0.939 (0.758, 1.162)	0.562	1	1.120 (0.704, 1.782)	0.633	1	1.081 (0.874,1.339)	0.472
Source of control	PB	3	0.855 (0.645, 1.134)	0.278	3	0.458 (0.183, 1.148)	0.096	4	0.749 (0.559, 1.005)	0.054	3	0.514 (0.248, 1.063)	0.072	3	0.676 (0.417, 1.095)	0.112
	HB	4	0.853 (0.470, 1.550)	0.603	4	0.902 (0.325, 2.507)	0.843	4	0.868 (0.439, 1.714)	0.683	4	0.944 (0.510, 1.748)	0.855	4	0.932 (0.586, 1.481)	0.765
Genotyping	mic-Array	2	0.908 (0.655, 1.257)	0.560	2	0.889 (0.581, 1.362)	0.590	3	0.884 (0.758, 1.030)	0.115	2	0.931 (0.752, 1.153)	0.511	2	0.955 (0.769, 1.185)	0.673
	PCR-RFLP	5	0.788 (0.508, 1.222)	0.287	5	0.561 (0.235, 1.336)	0.192	5	0.691 (0.412, 1.158)	0.161	5	0.617 (0.314, 1.212)	0.161	5	0.744 (0.482, 1.147)	0.181
Sample size	<500	4	0.861 (0.443, 1.677)	0.661	4	0.724 (0.203, 2.580)	0.619	4	0.808 (0.372, 1.758)	0.591	4	0.739 (0.297, 1.836)	0.515	4	0.827 (0.453, 1.510)	0.537
	≥500	3	0.849 (0.666, 1.083)	0.187	3	0.620 (0.294, 1.308)	0.210	4	0.804 (0.643, 1.005)	0.055	3	0.696 (0.358, 1.353)	0.285	3	0.791 (0.542, 1.154)	0.224
Quality score	<8	4	0.853 (0.470, 1.550)	0.603	4	0.902 (0.325, 2.507)	0.843	4	0.868 (0.439, 1.714)	0.683	4	0.944 (0.510, 1.748)	0.855	4	0.932 (0.586, 1.481)	0.765
	≥8	3	0.855 (0.645, 1.134)	0.278	3	0.458 (0.183, 1.148)	0.096	4	0.749 (0.559, 1.005)	0.054	3	0.514 (0.248, 1.063)	0.072	3	0.676 (0.417, 1.095)	0.112

Among the different methods that were used to genotype samples, the mic-array results showed that CC genotype or C allele of rs701848 was associated with increased cancer risk (Table 3). For sample sizes ≥ 500, individuals carrying CC, or combined TC/CC genotypes, or C allele in the rs701848 SNP were associated with increased cancer risk (Table 3). In studies with quality score ≥ 8, we observed association between rs701848 SNP and cancer risk in all models except heterozygous model (Table 3). The results of subgroup analyses by genotyping and sample size showed no significant association between rs2735343 polymorphism in PTEN with cancer risk (Table 4).

### Meta-regression analysis

We conducted meta-regression analysis based on ethnicity, cancer type, source of controls, genotyping methods, quality score (at the median cut-off point of 8), and sample size parameters to determine the factors that are critical for association of the *PTEN* SNPs with cancer risk. The data showed that cancer type determined the association between rs701848 and cancer risk (P < 0.05), indicating that there exists genetic heterogeneity between different cancer types.

### Publication bias and sensitivity analysis

**Figure 5:**
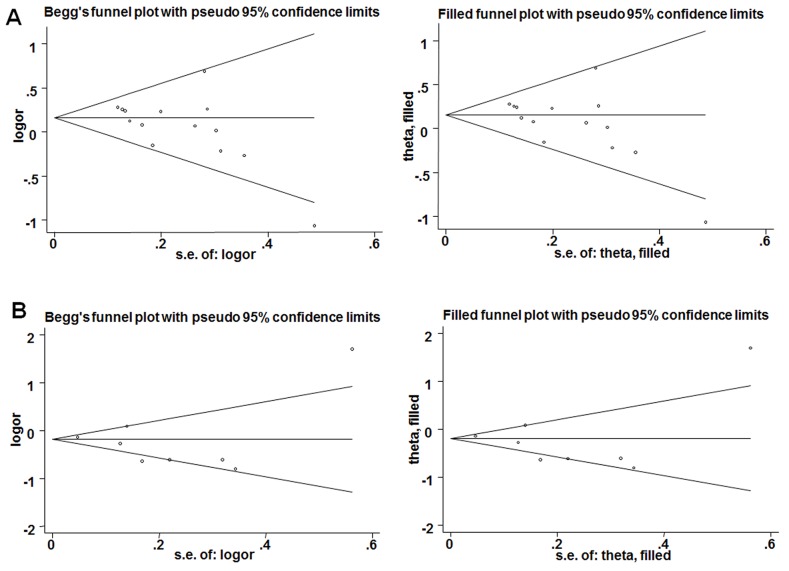
Results of Begg's tests **(A)** Begg's funnel plots (left) and filled funnel (right) plots for recessive model of rs701848; **(B)** A. Begg's funnel plots (left) and filled funnel (right) plots for dominant model of rs2735343.

We performed Egger's (Table 2) and Begg's (Table 2, Figure 5) tests to evaluate potential publication bias. The analysis showed no evidence of publication bias for all genetic models except homozygous and recessive models for rs701848. We conducted sensitivity analysis by Duval and Tweedie trim and fill method, which further confirmed that the results of this meta-analysis were statistically robust (Figure 5).

## DISCUSSION

PTEN exerts its tumor suppressor function by acting as a negative regulator of the mTOR/Akt signaling pathway [38]. Mutations in *PTEN* have been reported as prognostic factors in several cancers [14–17, 19, 39, 40]. Patients with homozygous intron 4 deletion in the *PTEN* gene are associated with increased risk of digestive tract cancer [41]. *PTEN* SNPs also play important roles in tumorigenesis. The *PTEN* rs11202586 SNP is associated with increased risk of testicular germ cell tumor [42]. Han *et al* showed that the *PTEN* rs3830675 SNP was associated with colorectal cancer in patients that consumed alcohol and smoked [43]. In this study, we systematically analyzed if rs701848 and rs2735343 SNPs increased cancer susceptibility. Our results indicated that CC genotype or C allele of rs701848 and GG genotype of rs2735343 increased the risk of cancer in Asian subjects.

Both rs701848 and rs2735343 SNPs are located in the intron and non-coding region of *PTEN* gene and increase cancer risk by probably influencing splicing, protein expression and cell cycle [44]. The rs701848 polymorphism influences cancer susceptibility by altering PTEN expression and reducing *PTEN* mRNA stability [29]. Although these functional genetic polymorphisms of *PTEN* were known to participate in tumorigenesis, their relationship with cancer risk was unknown [1–3, 45, 46]. Jang *et al* [23] and Xu *et al* [29] showed that C allele of rs701848 was more susceptible than the T allele in developing ESCC. In our study, we investigated 5458 cancer cases and 6003 controls and showed that the CC and CT genotypes or C allele of *PTEN* rs701848 SNP contributed to ESCC risk, especially, the individuals carrying CC genotype in PTEN rs701848 have a 1.813-fold increased cancer risk of ESCC. Our conclusion was different from Ma's study that investigated 206 ESCC cases and controls each and concluded that the rs701848 CC genotype was not associated with ESCC risk [24]. We performed subgroup analysis and did not find correlation between rs701848 and increased risk of other cancers [20, 21, 25, 33, 35]. Our results also showed that rs2735343 GG genotype was associated with increased cancer risk supporting Jang's [23] and Ma's [24] findings.

Cancer is a genetic disease because the underlying causes include somatic mutations, chromosome translocations, gene amplification, and epigenetic changes [47–49]. A single nucleotide polymorphism (SNP) can be a driver mutation in some cancer types. The accumulation of driver gene mutations are not synchronous and result in cellular heterogeneity within individual tumors [50]. Therefore, genetic heterogeneity is a distinguishing criterion for many cancer types. We comprehensively explored possible origins of heterogeneity by both sub-group and meta-regression analyses and demonstrated that in most genetic models our overall analyses was robust and consistent.

The major drawback of our meta-analysis was that it limited to individuals of Asian descent. Therefore, the effects of rs701848 and rs2735343 on non-Asian populations need to be studied in well-designed and large scale case-control studies. In conclusion, we demonstrate that the C allele of rs701848 and G allele of rs2735343 in PTEN gene increases cancer risk in Asian populations.

## MATERIALS AND METHODS

### Literature search strategy

This meta-analysis was performed according to the protocols of the *Observational Studies in Epidemiology (MOOSE) group* [51]. Two researchers, SDD and ZQ, independently searched the PubMed, EMBASE, Cochrane library, and Chinese National Knowledge Infrastructure (CNKI) databases for potentially eligible studies until March 31, 2017 without any language restrictions. The following combination of subjects and words were used for the searches: (“rs701848” or “rs2735343” or “polymorphism” or “variants” or “SNP”) and (“PTEN” or “phosphatase and tensin homolog”) and (“cancer carcinoma” or “tumor” or “tumour” or “cancer” or “cancer neoplasms” or “malignancy”). We excluded articles not meeting our eligibility criteria by screening titles and abstracts. Then, we screened the full text articles manually to identify all published studies that analyzed the relationship of *PTEN* rs701848 or rs2735343 with cancer risk.

### Inclusion criteria

The inclusion criteria for eligible articles were as follows: (1) the articles assessed the association between rs701848 or rs2735343 and cancer risk; (2) it was a case-control study; (3) study subjects diagnosed with malignant tumors were histologically confirmed; (4) sufficient data was available to calculate OR and the corresponding 95% CI. When the data in the articles was insufficient, we attempted to obtain the missing data from the first or corresponding authors via email.

### Data extraction and quality assessment

As mentioned above, two reviewers, SDD and LJH independently searched articles, extracted data and assessed the quality. If there was a controversy, a third researcher, ZQ, was involved to resolve the issue by discussion. The extracted data included first author, published year, country of origin, ethnicity, cancer type, genotyping method, characteristics of cases and controls, source of controls, and P value for HWE. The quality of studies was assessed by the Newcastle-Ottawa quality assessment scale for observational studies. The assessment scale had three categories, namely, selection, comparability, and exposure, which altogether contained eight items. A study was awarded a maximum of one point for each parameter within the selection and exposure categories. A maximum of two points were awarded for comparability. The maximum obtainable score was nine.

### Statistical analysis

The control group was analyzed by chi-square test and P > 0.05 was in accordance to HWE [52]. The relationship between rs701848 or rs2735343 and cancer risk was analyzed by ORs with 95% CIs in recessive (CC vs. CT+TT), dominant (TC+CC vs. TT), homozygous (CC vs TT), heterozygous (TC vs TT), and additive (C vs. T) models for rs701848 and homozygous (CC vs. GG), heterozygous (GC vs. GG), dominant (GC+CC vs. GG), recessive (CC vs. GC+GG) and additive (C vs G) models for rs2735343, respectively. Raw genotype frequency data was used to calculate the study-specific estimates of the OR without adjustments. The significance of the differences between cancer and study subjects was determined by performing Z test of pooled ORs, and P < 0.05 was considered significant. Heterogeneity analysis was tested among studies using *I*^2^ test. A *I*^2^ > 50% suggested heterogeneity [53]. A random-effects model was used if there was significant heterogeneity; otherwise, fixed-effect model was chosen for analysis. When there was significant heterogeneity, meta-regression and subgroup analyses were performed according to ethnicity, cancer type, source of controls, genotyping methods, quality score (at the median cut-off point of 8), and sample size [54]. The Taqman, SNPscan, multiplexed bead array, and Infinium methods of genotyping were classified as mic-Array for subgroup-analysis. Sensitivity analysis was assessed by trim and fill method to evaluate the reliability and stability of the meta-analysis results [18]. Publication bias was assessed qualitatively by funnel plots and quantitatively by Begg's [55] and Egger's [56] tests, respectively. A *P*< 0.05 for Begg's and Egger's tests indicated significant publication bias. Data were analyzed using STATA 12.0 (Stata Corporation: College Station, TX, USA) and Review Manager 5.3 (Copenhagen: Nordic Cochrane Centre, the Cochrane Collaboration, 2014) software.

## SUPPLEMENTARY MATERIALS FIGURES AND TABLES


