# Supplementary material for: Single nucleotide polymorphisms rs701848 and rs2735343 in PTEN increases cancer risks in an Asian population
Source: Oncotarget. 2017 Oct 24;8(56):96290–300. doi: 10.18632/oncotarget.22019 (PMC5707100; doi:10.18632/oncotarget.22019)
Supplement: Supplementary file 1 [file oncotarget-08-96290-s001.pdf]

Single nucleotide polymorphisms rs701848 and rs2735343 in PTEN increases cancer risks in an Asian population

SUPPLEMENTARY MATERIALS

Supplementary Table 1: Newcastle-Ottawa quality assessment scale for each included study

Studies		Selection				Comparability		Exposure			
	Year	Case definition adequate	Representativenes of the cases	Selection of controls	Definition of controls	Adjustment for age	Adjustment for lifestyle/ traditional risk factors	Ascertainment of exposure	Uniform method of ascertainment	Non-response rate	Total quality score
Li	2017	1	1	1	1	0	0	1	1	1	7
Chen	2016	1	1	1	1	0	0	1	1	0	6
Lin	2015	1	1	1	1	1	1	1	1	0	8
Xu	2015	1	1	1	1	1	1	1	1	0	8
Jing	2014	1	1	1	1	1	1	1	1	0	8
Jang	2013	1	1	1	1	1	1	1	1	0	8
Ma	2012	1	1	1	1	1	1	1	1	0	8
Cao	2012	1	1	1	1	1	1	1	1	1	9
Chen	2012	1	1	1	1	1	1	1	1	1	9
Slattery	2012	1	1	1	1	1	1	1	1	1	9
Ding	2011	1	1	1	1	1	1	1	1	0	8
Lacey	2011	1	1	1	1	1	0	1	1	1	8
Hiroshi	2009	1	1	1	1	1	0	1	1	0	7
Song	2009	1	1	1	1	0	0	1	1	0	6
Liu	2009	1	1	1	1	0	0	1	1	0	6
Shi	2009	1	1	1	1	0	0	1	1	0	6
Liu	2008	1	1	1	1	0	0	1	1	0	6
Rajaraman	2007	1	1	1	1	1	0	1	1	0	7
